# Evaluation and Treatment of Dysphagia in Public and Private Intensive Care Units (ICUs) in Greece

**DOI:** 10.1055/s-0043-1767676

**Published:** 2024-02-05

**Authors:** Soultana L. Papadopoulou, Evangelia Kitsanou, Ermioni Brahimi, Georgios Papathanakos, Ioannis Andrianopoulos, Stavroula J. Theodorou, Vasilios Koulouras, Nafsica Ziavra

**Affiliations:** 1 Department of Speech and Language Therapy, School of Health Sciences, University of Ioannina, Ioannina, Greece; 2 Intensive Care Unit, University Hospital of Ioannina, Ioannina, Greece; 3 Department of Radiology, University Hospital of Ioannina, Ioannina, Greece

**Keywords:** dysphagia, critical care, intensive care units, survey

## Abstract

**Introduction** Dysphagia is a significant but underrecognized clinical issue in the intensive care unit (ICU), and it is associated with various complications. Despite its clinical importance, there is limited research and no Greek ICU-specific guidelines for managing dysphagic patients. Additionally, only a few ICUs in Greece have dysphagia specialists, specifically speech-language pathologists (SLPs) providing their expertise.

**Objective** Τo identify the current practices for dysphagia management (screening, assessment, treatment) and gain insight into ICU directors' awareness/perceptions of the prevalence, complications, and risk of dysphagia.

**Materials and Methods** We identified 138 Greek ICUs. Data were collected from ICU (including pediatric and neonatal) directors, working in public and private hospitals, via a 24-item, anonymous online questionnaire, within a 4-month period.

**Results** Our survey was completed by 45 ICU directors. Most participants (84.4%) reported that dysphagia is a relevant clinical problem in their ICU, and 51.1% estimated a frequency rate < 20%. Non-instrumental approaches are mainly utilized to screen and diagnose dysphagia, whereas enteral nutrition and diet modifications are used to manage dysphagia. Additionally, 64.4% of ICU directors agreed that SLPs are essential for the management of dysphagic patients, and 66.7%, that awareness of dysphagia in their ICU could be increased.

**Conclusion** The current study documented the methods and approaches used to manage dysphagic patients in Greek ICUs. The ICU directors seem to recognize the clinical significance of dysphagia and its complications. According to our findings, the employment of SLPs could result in a more comprehensive and intensive approach and improve the quality of care for these patients.

## Introduction

Dysphagia, swallowing disorder, and deglutition disorder/dysfunction are terms that are frequently used interchangeably to describe any difficulty or inability to effectively and safely transfer liquids, food, saliva, and medicines from the mouth to the esophagus, during the oral preparatory, oral transit, pharyngeal, and esophageal stages of swallowing. 1 Since swallowing disorders are increasingly observed among patients in intensive care units (ICUs), 2 the term ICU-acquired swallowing disorder was introduced, suggesting multiple potential pathomechanisms in critical illness that lead to acquired dysphagia. 3 Most ICU patients require endotracheal intubation, which is considered a major risk factor for dysphagia in this population; therefore, the term postextubation dysphagia (PED) is also increasingly used. 4 Postextubation dysphagia is usually an ICU-acquired disorder. However, because dysphagia can present insidiously and have varied diagnostic criteria, a critical illness may unmask a previously undiagnosed swallowing disorder. 3

In a systematic review, Skoretz et al. 5 reported a prevalence of PED in the critical care setting ranging from 3% to 62%, with most cohort studies reporting an incidence greater than 20%. More recent data from a large-scale prospective study 6 in a non-selected ICU population revealed a 12.4% incidence of PED in the ICU, with dysphagia mostly persisting until hospital discharge. Although the underlying mechanisms of dysphagia in critically-ill patients remain incompletely understood, 7 the etiology is considered multifactorial. 3 7 8 9 10 11 Dysphagia can lead to various medical complications, such as aspiration pneumonia 12 and malnutrition, 13 which are noticed daily in many critically-ill patients, 7 and have been associated with compromised patient outcomes, such as delayed return to oral intake 10 14 and a higher mortality rate, 6 12 14 15 among others. 3 4 5 16 17 18

Considering the serious clinical consequences of dysphagia, timely and systematic screening of all critically-ill patients is necessary, to enable the early identification of dysphagia and, hopefully, the prevention of at least some of its complications. 19 The benefits of dysphagia screening have been well documented in the literature for decades. 20 21 However, systematic screening for dysphagia is uncommon in most ICUs, with screening methods mostly deriving from those applied to stroke patients, 6 while few screening tools have been studied in ICU patient populations. 22 23 24

For the timely assessment of dysphagia in critically-ill patients, both non-instrumental and instrumental measures are available. Non-instrumental assessments are usually performed by trained dysphagia specialists, such as a speech-language pathologist (SLP), a physiotherapist, or, in some cases, an occupational therapist. 1 6 19 25 The most common diagnostic test for PED is a bedside swallow evaluation (BSE) performed by an SLP. 3 However, its inability to rule out aspiration and provide objective information on pharyngeal swallow function are major drawbacks. 26 The fiberoptic endoscopic evaluation of swallowing (FEES) and the videofluoroscopic swallow study (VFSS) are the two instrumental evaluations of swallowing considered reference standards for dysphagia evaluation. 27 Since these assessments are complementary, once for example a patient has undergone FEES, a referral to complete a VFSS remains a viable option, and vice versa. 28

The development and implementation of a patient evaluation protocol as well as some, sometimes quite simple, interventions can help prevent complications, as well as improve the prognosis and comfort of ICU patients. 29 Acute-stroke populations also dominate the evidence base for dysphagia treatment in ICUs. Unfortunately, there is limited research on dysphagia interventions in critical-care settings and limited evidence to guide the clinical practice in this area at present. 30

The ICU multidisciplinary team (MDT) has evolved from a traditional medical and nursing model to encompass a growing allied health workforce including SLPs. 31 Despite the variation across ICUs in decisional responsibility and dysphagia referral pathways, 32 33 34 35 36 37 38 this multidisciplinary model of care has been suggested to improve patient outcomes. 2 39 40 41 42 The value of SLPs as integral members of the ICU MDT is becoming increasingly recognized worldwide, 43 44 45 46 and has been demonstrated in the literature. 25 26 36 39 47 48 49 50 51 52 53 In Greece, only about 5% of public hospitals have a permanent SLP on staff, resulting in a significant knowledge to practice gap regarding critically-ill patients, who remain intubated in ICU and post-ICU settings for an extended period without proper dysphagia referral and management options. 54 Furthermore, despite this condition's clinical importance, there is little research and no national ICU-specific guidelines to manage this population.

Therefore, we conducted a national survey to determine the current standard of care for dysphagia in Greek ICUs – specifically: a) the current practices for dysphagia management (screening, assessment/diagnosis, and treatment) and b) clinicians' awareness/perceptions of the prevalence, complications, and risk of dysphagia – to guide subsequent research and establish a basis for future nationwide diagnostic and management standards.

## Materials and Methods

### Survey Design

The research team conducted an online cross-sectional survey. We opted for a non-probability sampling technique, specifically purposive sampling, to identify and select individuals who are knowledgeable and experienced with our study's phenomenon of interest, dysphagia in the ICU. The ICU directors in Greece are senior doctors specialized in intensive care medicine who have both clinical and administrative responsibilities. Thus, all ICU directors working in non-coronavirus disease 2019 (Covid-19) ICUs, including pediatric ICUs (PICUs) and neonatal ICUs (NICUs), in public and private hospitals in our country, were eligible to participate. More specifically, the inclusion criteria were availability and willingness to participate, whereas the exclusion criteria were unavailability or unwillingness to participate and ICU directors working in Covid units, since it is still unknown if Covid patients in Greece present with dysphagia.

**Fig. 1:**
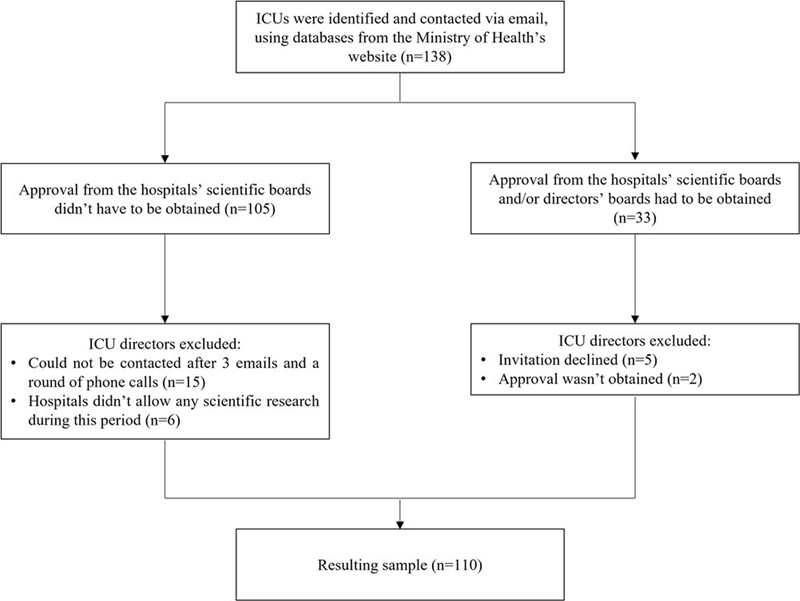
Recruitment process of the present study.

Initially, 138 ICUs were identified and contacted via email, using databases from the Ministry of Health's website. The resulting sample consisted of 110 ICU directors. More details about the recruitment process are presented in Figure 1 . The written survey was sent via email, to ensure participant anonymity, to maximize respondents' convenience (e.g., directors could choose when to fill out the survey, and there was no set time frame for completion such as in in-person surveys), and, most importantly, to protect both the participants' and the research teams' health during the Covid-19 pandemic.

### Questionnaire

A study-specific questionnaire, comprised of 24 closed-ended questions, was designed based on the current literature 34 35 37 38 and the professional and clinical experience of the authors. Emphasis was given to the development of clear and precise questions, tailored to our target population's level of knowledge on the topic, by using terms respondents could easily understand, and by avoiding words with vague or ambiguous meanings. We made certain our questions were phrased neutrally, with no bias towards one answer or another.

To ensure that the survey was understood by respondents as intended by the research team, the questionnaire was reviewed through a cognitive testing interview. Each questionnaire item was read aloud to an independent intensivist with more than ten years of clinical experience, who was asked to think aloud and describe their thought process as they answered the questions. Based on their feedback, minor wording changes were made to enhance the participants' comprehension.

The final 24-item questionnaire required about 10 minutes to be filled out, and included 4 questions regarding ICU characteristics/demographics, as well as 20 questions divided into the following 3 domains:1) clinicians' awareness/perceptions about dysphagia prevalence, risk factors, and complications (8 questions), 2) dysphagia screening and assessment (8 questions), and 3) dysphagia management/treatment in the ICU and SLP involvement (4 questions).

The chosen survey administration software was Google Forms, and single-answer as well as multiple-answer multiple-choice questions were used. To enable the anonymous completion of our survey, turning on the option “Allow only one response per user.” to prevent “multiple participation” of responders wasn't applicable, since it would require respondents to sign in with their Google account to access the form. The responders were instructed to fill out the survey only once. However, to detect any possible duplicates, we used the COUNTIF function in Google Sheets: =IF(COUNTIFS($A$2:$A$10,$A2,$B$2:$B$10,$B2,$C$2:$C$10,$C2…) > 1, “Duplicate”,””).

Ultimately, the questionnaire was distributed between February and June 2022, via email, to 110 Greek ICU directors, with responses being collected until July 2022. Three email reminders were sent to all participants at four-week intervals, whereas a final inquiry was made once more three weeks later, to further increase study participation.

To ensure accurate and complete reporting of our study, we used the Consensus-Based Checklist for Reporting of Survey Studies 55 (CROSS).

### Statistical Analysis

Qualitative and demographic data were presented as absolute and relative frequencies (percentages), while data from multiple-response questions were presented as absolute frequencies and percentages of cases. Data were displayed using frequency tables. The descriptive statistical analysis was performed using the IBM SPSS Statistics for Windows (IBM Corp., Armonk, NY, US) software, version 28.0.

### Ethical Considerations

The survey was filled out anonymously. None of the respondents were compensated for their involvement, and participation was entirely voluntary. A consent form, on the second page of the survey, had to be signed, by selecting the option “agree”, before participants could begin filling out the questionnaire.

Our study was approved by the Research Ethics Committee at of the University of Ioannina in March 16, 2022. Furthermore, in accordance with national law and regulations, ethical approval from the first, sixth, and seventh health districts was obtained.

## Results

### ICU Characteristics

By the end of the survey period, data were collected from 45 ICU directors. The overall response rate was 40.9%. More specifically, 37 (82.2%) participants worked in public hospitals, 43 (95.6%) units were adult units (ICUs), and most ICUs were multifunctional (medical/surgical: n = 42; 93.3%; Table 1 ). The responding units varied in terms of size (number of total beds per unit) and number of patients treated per year, with 5 to 10 beds (n = 19; 42.2%) and 200 to 400 patients (n = 17; 37.8%) being the most common, respectively ( Table 1 ).

**Table 1:** ICU characteristics

		N	%
ICU type	ICU	43	95.6
	PICU	1	2.2
	NICU	1	2.2
	Public hospital	37	82.2
	Private hospital/clinic	8	17.8
	Multifunctional (medical/surgical)	42	93.3
	Surgical	1	2.2
	Other	2	4.4
Number of beds	1–5	3	6.7
	5–10	19	42.2
	10–15	9	20.0
	15–20	8	17.8
	20–25	2	4.4
	> 25	4	8.9
Patients hospitalised per year	< 200	12	26.7
	200–400	17	37.8
	400–600	8	17.8
	600–800	2	4.4
	800–1,100	4	8.9
	1,100–1,400	1	2.2
	> 2,000	1	2.2
	Total	45	100.0

*Abbreviations: ICU, Intensive Care Unit; PICU, Pediatric Intensive Care Unit; NICU, Neonatal Intensive Care Unit* *.*

### Dysphagia in the ICU

As shown in Table 3 , 84.4% (n = 38) of the ICU directors reported that dysphagia is a relevant clinical issue in their unit. A total of 23 (51.1%) participants estimated a dysphagia frequency rate lower than 20%, and 42 (93.3%) stated that dysphagia most commonly occurs during the pharyngeal phase of swallowing ( Table 3 ).

Preexisting (n = 32; 71.1%) and acute neurological conditions (n = 37; 82.2%) were considered major risk factors for dysphagia ( Table 2 ). As Table 4 shows, 27 (60.0%) participants consider dysphagia a factor associated with prolonged ICU stays. Dysphagia was also identified as a risk factor for mortality by 21 (46.7%) ICU directors. Regarding the question of whether dysphagia increases hospital expenditures (total in-hospital treatment costs), 30 (66.7%) directors answered “yes”. The medical complication most often observed in the ICU, due to dysphagia, was aspiration pneumonia (n = 40; 88.9%; Table 2 ).

**Table 2:** Dysphagia risk factors

		N	% of cases
Major dysphagia risk factors	Age	24	53.3
	Emergency ICU admission	7	15.6
	Metabolic disorder	5	11.1
	Acute neurological conditions	37	82.2
	Trauma	33	73.3
	Sepsis/septic shock	12	26.7
	Preexisting or ICU-related sarcopenia	36	80.0
	Preexisting neurological conditions	32	71.1
	Oral/nasotracheal intubation	21	46.7
	Long-term intubation	33	73.3
	Nasogastric feeding tube	13	28.9
	Sedatives and muscle relaxants	20	44.4
	Opioids	13	28.9
	Neurotropic medication	6	13.3
	Total	292	648.9

*Abbreviation: ICU, Intensive Care Unit.*

### Dysphagia Evaluation (Screening and Assessment)

Regarding the implementation of a protocol to diagnose and treat dysphagia, 20 (44.4%) ICUs did not have a standard of care (SOC). Additionally, 15.6% (n = 7) of the ICUs systematically screen every patient. Most units (n = 30; 66.7%) perform screening tests systematically, on an individual basis, and more specifically, mainly on patients with clinical signs of dysphagia, such as aspiration (n = 16; 35.6%; Table 5 ).

**Table 3:** Dysphagia in Intensive Care Units

Frequency tables				
		N	%	% of cases
Dysphagia is a clinical problem in the Intensive Care Unit	Yes	38	84.4	−
	No	3	6.7	−
	Maybe	4	8.9	−
Estimated frequency rate	< 20%	23	51.1	−
	20–40%	10	22.2	−
	40–60%	9	20.0	−
	60–80%	2	4.4	−
	80–100%	1	2.2	−
	Total	45	100.0	−
Phase in which dysphagia occurs	Oral preparatory phase	10	−	22.2
	Oral phase	28	−	62.2
	Pharyngeal phase	42	−	93.3
	Esophageal phase	6	−	13.3
	Total	86	−	191.1

**Table 4:** Dysphagia-related complications

		N	%	% of cases
Dysphagia-related complications	Aspiration pneumonia	40	−	88.9
	Sepsis	24	−	53.3
	Re-admission to the Intensive Care Unit	18	−	40.0
	Re-intubation	26	−	57.8
	Decannulation/extubation failure	14	−	31.1
	Need for tracheostomy	27	−	60.0
	Malnutrition/dehydration	17	−	37.8
	Depression	5	−	11.1
	No dysphagia-related complications	3	−	6.7
	Total	174	−	386.7
Dysphagia prolongs stay in the Intensive Care Unit	Yes	27	60.0	−
	No	8	17.8	−
	Maybe	10	22.2	−
Dysphagia increases hospital expenditures	Yes	30	66.7	−
	No	9	20.0	−
	Maybe	6	13.3	−
Dysphagia increases mortality rates	Yes	21	46.7	−
	No	12	26.7	−
	Maybe	12	26.7	−
	Total	45	100.0	−

**Table 5:** Dysphagia screening

		N	%
Dysphagia protocol	Yes	19	42.2
	No	20	44.4
	Planning to implement in near future	6	13.3
Systematic screening	Yes, all patients	7	15.6
	Yes, individual basis	30	66.7
	No	6	13.3
	Planning to perform screening tests in near future	2	4.4
Patients screened	All patients	7	15.6
	After extubation	12	26.7
	Preexisting dysphagia	2	4.4
	Clinical signs of dysphagia	16	35.6
	No screening	8	17.8
Screening method	Food trials	2	4.4
	Water swallow test	19	42.2
	Bedside swallow evaluation	21	46.7
	Fibereoptic endoscopic evaluation of swallowing	1	2.2
	Videofluoroscopic swallow study	2	4.4
	Total	45	100.0

As shown in Table 6 , the specialist responsible for the initial dysphagia screening appears to differ from ICU to ICU. Among various disciplines, intensivists (n = 30; 66.7%) were reported with the highest frequency as the experts responsible for this testing, either by themselves or in collaboration with other disciplines/as part of an MDT. For the number of selected answers/choices and their frequency, see Table 6 . Two non-instrumental methods are mainly used to screen for dysphagia in the ICU: the water swallow test (WST) (n = 19; 42.2%) and the BSE (n = 21; 46.7%; Table 5 ).

**Table 6:** Healhcare providers in charge of dysphagia evaluation (screening and assessment)

		N	% of cases	Number of selected choices	N	%
Screening performed by:	Trained Intensive Care Unit nurse	8	17.8	1	22	48.9
	Any nurse	8	17.8			
	Intensivist	30	66.7	2	18	40.0
	Ear, nose, and throat physician	9	20.0			
	Speech-language pathologist	8	17.8	3	4	8.9
	Physical therapist	8	17.8			
	No screening	1	2.2	4	1	2.2
	Other	2	4.4			
	Total	74	164.4	Total	45	100.0
Assessment (specialist examination) performed by:	Intensivist	30	66.7	1	25	55.6
	Ear, nose, and throat physician	16	35.6			
	Speech-language pathologist	8	17.8	2	17	37.8
	Physical therapist	1	2.2			
	Trained Intensive Care Unit nurse	10	22.2	3	3	6.7
	Any nurse	1	2.2			
	No specialist examination	2	4.4			
	Total	68	151.1	Total	45	100.0

As shown in Table 7 , 60.0% (n = 27) of the participants reported that their unit uses a sequential approach to evaluate dysphagic patients (screening followed by an assessment performed by a dysphagia specialist).

**Table 7:** Dysphagia assessment and treatment

		N	%	% of cases
Sequential evaluation approach	Yes	27	60.0	−
	No	18	40.0	−
Assessment (diagnostic) method	Clinical signs	11	24.4	−
	Clinical examination/Bedside swallow evaluation	21	46.7	−
	Fibereoptic endoscopic evaluation of swallowing	9	20.0	−
	Videofluoroscopic swallow study	3	6.7	−
	Other	1	2.2	−
	Total	45	100.0	−
Treatment/management methods	Nil per os	10	−	22.2
	Enteral nutrition	39	−	86.7
	Diet modifications	23	−	51.1
	Swallowing training	15	−	33.3
	Tracheostomy	15	−	33.3
	Total	102	−	226.7
Number of selected choices	1	12	26.7	−
	2	14	31.1	−
	3	14	31.1	−
	4	5	11.1	−
	Total	45	100.0	−

Similarly to the screening process, specialists perform dysphagia assessments either alone or in collaboration with other professionals. Specifically, as shown in Table 6 , among the specialists in charge of this assessment, testing by intensivists (n = 30, 66.7%) was mostly reported. For the number of selected choices and their frequency, see Table 6 . To diagnose ICU patients with dysphagia, clinicians mostly rely on non-instrumental approaches, such as the clinical examination/BSE (n = 21; 46.7% Table 7 ).

### Management of Dysphagia and the Presence of SLPs in the ICU

To treat dysphagic patients in the ICU, several methods are used ( Table 7 ), with enteral nutrition (n=39; 86.7%) being the one most often utilized. For the number of selected answers/choices and their frequency, see Table 7 .

Most ICUs (n = 34; 75.6%) participating in the present study did not have a dedicated/assigned SLP. Services by SLPs were available in 11 (24.4%) units. More specifically, in most ICUs (n = 5; 11.1%), SLPs were independent contractors. In these units, their main responsibilities were evaluating/assessing, and diagnosing dysphagic patients (n = 8; 72.7% Table 8 ).

**Table 8:** Involvement of speech-language pathologists and dysphagia awareness in Intensive Care Units

		N	%	% of cases
Speech-language pathologists working in the Intensive Care Unit	Yes	2	4.4	−
	No	34	75.6	−
	As a counsellor	4	8.9	−
	Independent contractor	5	11.1	−
	Total	45	100.0	−
Responsibilities of the speech-language pathologist	Assessment and diagnosis	8	−	72.7
	Treatment and management	5	−	45.5
	Counselling	8	−	72.7
	Total	21	−	190.9
Necessity of a speech-language pathologist in the Intensive Care Unit	Yes	29	64.4	−
	No	3	6.7	−
	Maybe	13	28.9	−
	Total	45	100.0	−
Specific domains in which the presence of a speech-language pathologist could be beneficial	Multidisciplinary approach	37	−	82.2
	Sharing responsibility	3	−	6.7
	Preventing serious medical complications	23	−	51.1
	Evaluation and diagnosis	20	−	44.4
	Management and treatment	32	−	71.1
	Counselling	19	−	42.2
	The presence of a speech-language pathologist would not be beneficial	4	−	8.9
	Total	138	−	306.7
Need for increased dysphagia awareness	Yes	30	66.7	−
	No	6	13.3	−
	Maybe	9	20.0	−
	Total	45	100.0	−

In total, 29 (64.4%) ICU directors agreed that SLPs are essential for the management of dysphagic patients. An SLP working in the ICU would be beneficial in domains such as the multidisciplinary approach to patients with dysphagia (n = 37; 82.2%; Table 8 ). Finally, 30 (66.7%) directors stated that dysphagia awareness in their ICU could be increased ( Table 8 ).

## Discussion

The current study is a cross-sectional online survey. We found that most directors recognized dysphagia as a significant clinical problem in their ICU. However, only a few ICUs systematically screen every patient and have a dysphagia protocol. Both screening and assessment are usually performed by an intensivist, either alone or in collaboration with other clinicians, through non-instrumental approaches, while enteral nutrition and diet modifications are commonly used to manage dysphagia. Furthermore, most ICU directors agreed that SLPs are essential for managing/treating dysphagic patients, and that awareness of dysphagia in their ICU could be increased.

As per the literature, the participants seem aware of dysphagia in terms of prevalence, 3 5 6 34 35 risk factors, 7 8 9 10 11 and dysphagia-related medical complications. 3 4 6 7 14 It has been demonstrated that many ICUs do not seem to address PED per protocol in the clinical practice, and there is no standardized approach to screening and/or assessment. 18 An online survey 32 of 528 respondents from 69 countries showed that only 28% of ICUs use a specified dysphagia-related protocol. However, Zuercher et al. 35 found that approximately 68% of Swiss institutions have a standard operating procedure (SOP) for oropharyngeal dysphagia. We found similar results, but with a lower reported frequency. A standardized dysphagia protocol, implemented by professionals specialized in dysphagia, could help identify patients with or at risk of dysphagia promptly, enabling more timely and efficient interventions, and potentially improving patient outcomes. 29 33 51

Several surveys have reported that only a few hospitals use a standardized dysphagia screening protocol for all patients, 32 37 have a standardized protocol that defines which patients should be assessed for dysphagia, 33 or screen all ICU patients. 35 We found similar results. As Brodsky et al. 18 suggested, differences in the awareness of dysphagia in the ICU may exist among patient populations. Clinicians in the ICU may be more sensitive to a higher prevalence of dysphagia among stroke patients or those with neurological conditions in general because decades of research have established a high prevalence and a high level of clinician awareness regarding this patient group. Surprisingly, no unit reported the systematic screening of patients with neurological conditions, possibly because this population is mostly admitted to step-down ICUs or other units in Greece. Increased awareness may also apply to patients with a tracheostomy, 41 44 51 or patients postextubation. 37 54 These groups were also screened with the highest frequency in Swiss ICUs. 35 This also seems to be the case in our country, since two patient groups were mostly screened: patients with clinical signs of dysphagia and patients postextubation.

According to a survey where 801 SLPs certified by the American Speech-Language-Hearing Association (ASHA) working in ICUs in the United States participated bedside screening protocols (such as a 3-ounce WST) were used by 41% of the hospitals. These screening protocols were most often administered by nursing staff (66%), followed by SLPs (27%). 37 In Greece, non-instrumental approaches (such as the WST, BSE) were mostly used. These results extend previous findings. 33 34 35 It should be noted that the BSE has been validated in ICU patients, 23 and that a study 22 on the validation of a dysphagia screening protocol that uses the 3-ounce WST demonstrated a sensitivity of 81% and a specificity of 69% in ICU patients. In the United States and Switzerland, nurses are mainly responsible for the initial dysphagia screening. 35 37 This does not agree with our findings. Because SLPs may be available only during standard weekday working hours, professionals from other disciplines, usually nurses, may have to provide the initial screening for dysphagia in the ICU. 22 Nonetheless, further research is needed to determine whether screening performed by nurses 48 or other health care providers would result in more expedient and more appropriate referrals to dysphagia specialists, such as SLPs, due to swallowing dysfunction.

In the United States and Switzerland, the diagnosis of dysphagia is usually established by an SLP using the BSE. 3 35 In a study in Australia, 38 the top reported assessment was the VFSS (79%), whereas a recent international survey 33 reported that the most common method used to confirm the presence of dysphagia was the WST (46%). Similarly to our findings, only a minority (8%) of ICUs used instrumental assessments. Additionally, the FEES seems to be the gold standard in most Dutch ICUs for the definitive assessment of swallowing function, with 60% occasionally using it in the screening work-up. The VFSS, on the other hand, was only used occasionally by 25% of the respondents. 34 This preference for the FEES, which we also reported, contrasts with practice in the United States, where the VFSS was available in 97% of the hospitals, while the FEES, in only 41%. 37 The decision to choose one instrumental assessment over the other may depend on the availability of the equipment to perform the procedure, the clinical questions that need to be answered, and/or clinician preference. The high prevalence of silent aspiration in this population, which goes undetected unless it has consequences, such as aspiration pneumonia or pneumonia, “unmask” its presence, supports the need for expert instrumental swallowing assessment, especially given the vulnerability of critically-ill patients. 20 56

Evidence for dysphagia treatment in critical-care patients is limited. 30 Macht et al. 37 found that treatment in ICUs usually focused on dietary texture modifications and postural changes/compensatory maneuvers, rather than on direct rehabilitation to improve swallowing function, such as neuromuscular electrical stimulation (NMES), which is also consistent with the findings of other studies. 36 38 In the survey by Spronk et al., 33 postural adjustments, with a frequency of 86%, were reported as a measure taken to prevent aspiration or aspiration pneumonia rather than a treatment modality for dysphagia. In comparison, in Swiss ICUs, dietary texture modification (78.4%) and swallowing training by dysphagia specialists, alongside functional therapy including postural changes (91.9%), were the most reported management techniques. Even though many patients in the ICU will have their nutritional and hydration needs met from the onset of their stay through enteral feeding, nasogastric feeding tubes, which are most often used, are considered a common risk factor for dysphagia in ICU patients; 29 therefore, their use as a dysphagia treatment modality, as was reported in our study, may be counterproductive.

In addition to the current study, in an international cross-sectional survey, 33 out of 746 ICUs, the Greek units that participated (n = 36) reported no SLP services available for this population. These findings highlight the small number of SLPs currently offering their expertise in Greek ICUs, which differs from international practice. 33 36 37 With focused training in feeding/swallowing anatomy and physiology, augmented by clinical knowledge and skills in domains such as voice, cognition, and communication, SLPs are uniquely qualified to excel in the critical-care setting. 52 Several national guidelines and position statements by various professional bodies specify the need for SLP involvement in the ICU, with a requirement for expertise, experience, and seniority to ensure that appropriate dysphagia assessments and interventions are delivered to critically-ill patients with complex conditions, including those requiring mechanical ventilation and tracheostomy. 43 44 45 46

Additionally, a recently published article by McRae et al. 51 aimed to increase awareness of the background training and skill development of SLPs working in this context and to demonstrate their range of specialist abilities. The article 51 illustrates the great value that SLPs add to the existing MDT in critical care, with their skills and expertise in swallowing, language, and communication. SLPs provide a range of assessments and interventions to enhance patient care, with opportunities for future development using advanced technologies. Internationally, SLP input in critical care is still limited though, in terms of dedicated posts, MDT involvement, consistent management approaches, and training opportunities. 36 Even if most critically-ill patients may not be ready for much direct assessment or intervention, the plan forward e.g., next steps toward oral feeding, could benefit from SLP guidance, in order to promote and protect any development or progress made. These discussions could provide wonderful opportunities for SLPs to educate team members regarding the SLP's role in the MDT ICU team and enable the presentation of research information or shared clinical experience to help nurses or other health care providers learn more about the SLP's perspective. 50

Since many Greek ICU directors are aware of the great value that SLPs could add to the existing ICU MDT, in various domains, a significant knowledge-to-implementation/clinical practice gap is evident. Malandraki et al. 54 suggested the need to increase awareness of the importance of early identification and treatment of dysphagia in critical-care settings in Greece, and to encourage additional training and specialization of health care professionals and advancement of the health care system so that these patients are promptly identified and treated. In this context, most participants agreed that dysphagia awareness in their ICUs should be increased.

Several limitations of the present study should be acknowledged. Firstly, our results are based on rough subjective estimates provided by individual respondents, not objectively-calculated data retrieved from hospital records. Secondly, the methods used to assess, evaluate, and manage/treat dysphagic ICU patients are likely the same for most physicians working in the same ICU, but some answers might still reflect individual preferences and expertise, possibly leading to a response bias. We believed that, since ICU directors oversee the clinical practice in the ICU, they would best represent the ICU team's view to complete the survey. However, other ICU healthcare professionals may perform other diagnostic or therapeutic techniques pertinent to the evaluation and management of dysphagia. A subsequent survey involving other ICU professionals, such as critical-care nurses, may be important to understand the assessment and management of this condition more thoroughly in our country. Thirdly, the number of responses we received was lower than anticipated, meaning that the responses may not be truly representative; therefore, caution with interpretation and generalizability is warranted. Furthermore, there were not enough respondents from private hospitals/clinics to examine the differences in dysphagia management between public and private ICUs.

## Conclusion

In the last decade, there has been an increased clinical but also research interest in dysphagia in the ICU. Dysphagia in critically-ill patients is a major clinical problem which can lead to adverse events and decrease these patients' quality of life. In the present study, we reported on the current practice patterns in Greek ICUs regarding screening, evaluation, and treatment/management of dysphagia. The ICU directors seem to recognize the clinical significance of dysphagia and its complications. However, most of the ICUs that participated in the present study systematically screen and assess patients only on an individual basis, mainly using non-instrumental approaches. Although the survey was conducted only in Greek ICUs, we do believe that the results are of interest both nationally, to guide subsequent research and establish a basis for future nationwide screening, diagnostic, and management standards, and internationally, as they can be compared to local practice and used for the consideration of possible alternative strategies. According to our findings, the employment of SLPs as well as an increase in dysphagia awareness among ICU health care professionals could help ICUs provide a more comprehensive, multidisciplinary, and intensive approach and improve the quality of care for these patients.
